# Patient Age Is Not a Determinant of 12-Month Pain Response After Autologous Adipose-Derived Stromal Vascular Fraction Injection for Knee Osteoarthritis

**DOI:** 10.3390/medicina62061044

**Published:** 2026-05-28

**Authors:** Yong Sang Kim, Yong Gon Koh

**Affiliations:** Center for Stem Cell & Arthritis Research, Department of Orthopaedic Surgery, Yonsei Sarang Hospital, Seoul 06073, Republic of Korea; yskimos@gmail.com

**Keywords:** knee osteoarthritis, stromal vascular fraction, age, body mass index, regenerative medicine, prognostic factors

## Abstract

***Background:*** Autologous adipose-derived stromal vascular fraction (SVF) is increasingly used for symptomatic knee osteoarthritis (OA), but it remains uncertain whether patient age should influence candidacy. We examined whether age was related to 12-month pain response after intra-articular SVF administration. ***Methods:*** This retrospective knee-level analysis included 357 knees from 266 patients with Kellgren-Lawrence grade II-IV OA treated with adipose-derived SVF and followed for at least 12 months. Pain was assessed with the visual analog scale (VAS). Group comparisons and Spearman correlation analyses were used to explore relationships between age, baseline variables, injected cell number, and pain outcomes. ***Results:*** VAS scores improved from 6.5 ± 1.2 before treatment to 3.1 ± 1.6 at final assessment (*p* < 0.01). Age did not show a significant association with baseline pain (*p* = 0.128), final pain (*p* = 0.088), or measured baseline factors. Higher body mass index, more severe radiographic OA, and lower SVF cell number were associated with less favorable final pain scores. No serious treatment-related adverse event was identified. ***Conclusions:*** SVF injection was followed by significant pain reduction at 12 months. In this cohort, chronological age was not a meaningful determinant of response, whereas metabolic burden, structural OA severity, and delivered cell dose were more relevant clinical factors. These results argue against excluding patients from SVF treatment solely because of age.

## 1. Introduction

Knee osteoarthritis (OA) is a progressive disorder of the entire joint, in which cartilage damage, subchondral bone change, synovial inflammation, and pain interact to impair daily activity [1,2,3]. The number of affected patients is rising as populations age and obesity becomes more common [4]. Common nonoperative treatments, such as oral non-steroidal anti-inflammatory drugs and intra-articular corticosteroid or hyaluronic acid injections, can reduce symptoms for some patients but have limited ability to alter the underlying degenerative process [4,5].

Biologic treatments have therefore been investigated as a way to improve the intra-articular environment rather than merely suppress symptoms [1,6]. Stromal vascular fraction (SVF) obtained from adipose tissue is a mixed autologous cell preparation that contains stromal cells, endothelial-lineage cells, pericytes, immune cells, and extracellular matrix-associated elements [1,7,8]. Its proposed benefit is not limited to direct tissue replacement; paracrine, anti-inflammatory, and immunomodulatory activities may also contribute to clinical improvement [4,7,9].

Existing clinical studies suggest that SVF injection can be performed safely and may provide pain relief in knee OA [9,10,11,12]. Nevertheless, the magnitude of benefit differs among patients, and the variables that best predict response remain debated. Recent investigations have therefore focused on identifying prognostic factors that may influence clinical outcomes following SVF therapy [1,13]. Selection of the appropriate patient is critical for optimizing the effectiveness of SVF therapy. The present analysis was designed to address a different question: whether chronological age is associated with 12-month pain outcome in a larger knee-level cohort, while also considering BMI, radiographic severity, and injected SVF cell number.

Among potential patient-related factors, age has traditionally been considered an important determinant of outcomes in many orthopedic and regenerative procedures [13]. Age is often viewed as a potential limitation for regenerative procedures because cellular senescence, lower proliferative capacity, impaired tissue repair, and advanced joint degeneration are more common in older individuals [14,15,16]. However, chronological age may not reflect the functional properties of the SVF product or the biological state of the knee joint. Since older adults constitute a major proportion of patients with knee OA, inappropriate age-based exclusion could restrict access to a potentially useful option. We therefore evaluated the association between age and clinical outcome after intra-articular autologous adipose-derived SVF injection. We hypothesized that age would not be significantly associated with pain improvement after treatment.

## 2. Materials and Methods

### 2.1. Study Design and Patient Selection

This retrospective cohort study was conducted in accordance with the Declaration of Helsinki and approved by the Institutional Review Board of Yonsei Sarang Hospital, Seoul, Republic of Korea (protocol code YSSR IRB 2026-03-001; date of approval: [11 March 2026]). Because only previously collected clinical data were analyzed, the requirement for informed consent was waived by the IRB. Consecutive patients treated with intra-articular autologous adipose-derived SVF for symptomatic knee OA between July 2024 and February 2025 were screened if they had completed at least 12 months of follow-up. Knees were eligible when OA was graded as Kellgren-Lawrence Kellgren-Lawrence (K-L) grade [17] II, III or IV and the diagnosis was supported by clinical examination, radiographs, and magnetic resonance imaging. Patients also had persistent knee pain and/or functional limitation despite at least 3 months of oral non-steroidal anti-inflammatory drug treatment. Exclusion criteria included steroid injection within the previous year, clinically relevant disease in the ipsilateral hip or ankle, hematologic or cardiovascular disease, systemic infection, immunosuppression, metabolic arthritis, joint infection, knee instability, varus or valgus malalignment of at least 5 degrees, and large meniscal tears.

### 2.2. SVF Harvesting, Processing, and Safety Checks

One day before injection, subcutaneous adipose tissue was obtained from the gluteal region using tumescent liposuction. Approximately 140 mL of lipoaspirate was collected from each patient and combined with sterile 1× phosphate-buffered saline. Samples were transported to the laboratory in sterile containers under controlled conditions. The cellular fraction was separated from mature adipocytes and connective tissue components by centrifugation (Hanil Scientific Inc., Gyeonggi-do, Republic of Korea) using a previously established SVF-processing workflow. For clinical use, 120 mL of the harvested adipose tissue was processed for injection. Before administration, the preparation underwent mycoplasma testing (iNtRON, Gyeonggi-do, Republic of Korea), endotoxin testing (Associates of Cape Cod, MA, USA), and Gram staining (BD Biosciences, Franklin Lakes, NJ, USA). Cell viability was measured by methylene blue dye exclusion (NanoEntek, Seoul, Republic of Korea). The remaining 20 mL was processed in parallel for laboratory characterization.

### 2.3. Laboratory Characterization of SVF

A colony-forming unit fibroblast (CFU-F) assay was used to confirm the presence of mesenchymal progenitor cells. SVF-derived cells were seeded at 16 cells/cm^2^ in T25 flasks and cultured under standard conditions. After incubation, colonies composed of at least 50 cells were counted by light microscopy [18,19]. For immunophenotyping, cells were expanded at 50 cells/cm^2^ until the cell number was sufficient for analysis. Fluorescence-activated cell sorting was used to measure CD14, CD34, CD90, and CD105 expression [20,21]. Approximately 2 × 10^6^ cells were allocated to each marker, resulting in a total requirement of 8 × 10^6^ cells for the four-marker assessment. Multilineage differentiation was assessed after adipose-derived stromal cells were plated at 5 × 10^3^ cells/cm^2^ in Dulbecco’s modified Eagle’s medium (DMEM; HyClone, Logan, UT, USA) with 10% fetal bovine serum (HyClone). After 24 h of adherence, cells were exposed to adipogenic, osteogenic, or chondrogenic induction media [22]. Differentiation was verified with lineage-specific histological staining.

### 2.4. Injection Technique and Post-Injection Instructions

After SVF preparation, the final SVF suspension was adjusted to a standardized volume of 2 mL per knee joint before intra-articular administration. For patients treated bilaterally, this volume was administered to each knee separately; therefore, the reported injection volume refers to each individual knee, not the total volume administered to both knees. A single experienced senior orthopedic surgeon performed all injections using a standardized intra-articular approach. Patients lay supine with the knee extended. If an effusion was present, aspiration was performed before injection. The SVF suspension was delivered through a transverse needle trajectory at the midpatellar level into the patellofemoral joint space while the patella was displaced superiorly and laterally [23]. Patients were instructed to avoid additional interventions, including physical therapy, acupuncture, steroid injection, and opioid or strong analgesic use, for 1 year after the procedure. For the first 3 days, they were also advised to avoid activities that placed excessive load on the treated knee, including prolonged standing, jogging, and heavy lifting.

### 2.5. Clinical and Radiographic Assessment

Pain was measured with the visual analog scale (VAS) at baseline and during follow-up. Safety was evaluated by recording adverse events. Radiographic evaluation consisted of weight-bearing anteroposterior, true lateral at 30 degrees of flexion, and hip-to-ankle standing anteroposterior radiographs. A musculoskeletal radiologist who was not involved in treatment and was blinded to the study purpose graded the radiographs using the Kellgren-Lawrence system [17]. Baseline radiographs were compared with images obtained at the final follow-up, approximately 12 months after SVF injection.

### 2.6. Statistical Analysis

The principal outcome variable was the difference between baseline and final follow-up VAS pain score. Continuous variables are expressed as means ± standard deviations unless otherwise stated. Because the treated knee was the unit of analysis, each knee was handled as an independent observation. Distributional normality was evaluated with the Shapiro-Wilk test. The Wilcoxon signed-rank test compared baseline and final VAS values. Fisher exact testing was used for categorical comparisons when appropriate. Relationships between baseline characteristics and pain outcomes were assessed with the Mann-Whitney U test or Kruskal-Wallis test. Because several clinical variables were ordinal or non-normally distributed, non-parametric statistical methods were used. Spearman rank-order correlation analysis was selected to evaluate monotonic associations between chronological age and baseline clinical variables without assuming normal distribution or linear relationships. The purpose of this analysis was exploratory and focused primarily on whether age was associated with pain outcomes or other baseline factors. A multivariable regression model was not performed because of the retrospective design, limited availability of potentially relevant confounding variables, and the exploratory nature of the present analysis. SPSS (Version 13.0, IBM Corp., Armonk, NY, USA) was used for all analyses, and *p* < 0.05 was considered statistically significant.

## 3. Results

### 3.1. Study Population

Of 278 screened patients, 7 discontinued follow-up and 5 were lost before final evaluation. The study population therefore consisted of 266 patients and 357 treated knees. Because the statistical unit was the treated knee, sex and side distributions were calculated per knee. The cohort included 111 male knees and 246 female knees. Mean age was 63.6 years (range, 43–86 years), and mean BMI was 25.7 kg/m^2^ (range, 18.9–32.1 kg/m^2^). Baseline features are listed in Table 1.

### 3.2. SVF Characterization

CFU-F testing demonstrated plastic-adherent fibroblast-like colonies, indicating that mesenchymal progenitor cells were present in the adipose-derived SVF preparations. Adipose-derived stromal cells accounted for approximately 9.8% of the SVF cell population.

The mean stromal cell count was 7.2 × 10^6^ cells, and the mean total injected SVF cell count was 7.3 × 10^7^ cells per sample (range, 6.5–8.5 × 10^7^ cells).

Flow cytometry showed strong expression of CD90 (99.2%) and CD105 (94.4%) and low expression of CD34 (5.24%) and CD14 (2.76%). SVF-derived cells also differentiated toward adipogenic, osteogenic, and chondrogenic lineages, as shown by lineage-specific staining.

### 3.3. Pain, Radiographic Findings, and Safety

Pain improved significantly over the follow-up period. The mean VAS score decreased from 6.5 ± 1.2 at baseline to 3.1 ± 1.6 at the final visit (*p* < 0.01). No serious adverse events were observed. Nine patients reported mild swelling with stiffness, and all cases resolved without further intervention.

At baseline, 143 knees (40.1%) were K-L grade II, 149 knees (41.7%) were grade III, and 65 knees (18.2%) were grade IV (Table 1). At 12 months, the corresponding numbers were 140 (39.2%), 150 (42.0%), and 67 (18.8%), respectively. This distribution did not differ significantly from baseline (*p* = 0.078).

### 3.4. Clinical Factors Associated with Pain Outcomes

Table 2 shows VAS scores according to baseline characteristics. Age categories were not significantly related to VAS score at baseline (*p* = 0.128) or final follow-up (*p* = 0.088). Sex and treated side were likewise not associated with pain outcomes.

Final pain scores differed significantly according to BMI, radiographic severity, and injected SVF cell-number category. Patients with BMI ≥30.0 kg/m^2^ had higher residual pain than patients in lower BMI categories (*p* < 0.001). K-L grade was associated with VAS score at both baseline (*p* = 0.003) and final follow-up (*p* = 0.001). Knees receiving fewer than 7.0 × 10^7^ SVF cells showed higher final pain scores than knees treated with larger cell numbers (*p* = 0.004).

Age was not significantly correlated with the measured baseline factors (Table 3): sex (rho = 0.097, *p* = 0.066), side of involvement (rho = 0.046, *p* = 0.384), BMI (rho = 0.090, *p* = 0.089), K–L grade (rho = −0.016, *p* = 0.770), or SVF cell number (rho = −0.064, *p* = 0.225). Thus, age was not a significant correlate of pain status, whereas BMI, OA grade, and cell dose were associated with final VAS outcome.

## 4. Discussion

This study investigated whether chronological age was associated with 12-month pain outcome after autologous adipose-derived SVF injection for knee OA. The main finding was that age did not significantly affect baseline or final VAS score. In contrast, BMI, K-L grade, and injected SVF cell number were related to final pain status. These data suggest that age alone is an insufficient basis for determining suitability for SVF treatment.

In the current study, patients demonstrated significant improvement in VAS pain scores at 12 months, which is consistent with the growing body of literature supporting the clinical efficacy of SVF therapy. Previous clinical investigations have reported meaningful reductions in pain and functional improvement following intra-articular SVF injection in knee OA patients [24]. Moreover, a recent systematic review concluded that SVF therapy generally provides favorable clinical outcomes with a good safety profile and minimal adverse events [4]. Our findings of significant pain reduction without serious complications further reinforce the safety and therapeutic potential of SVF as a regenerative treatment option for knee OA.

From a mechanistic standpoint, SVF contains a heterogeneous population of regenerative and immunomodulatory cells, including adipose-derived stem cells, macrophages, and pericytes, which collectively exert anti-inflammatory and paracrine effects within the osteoarthritic joint [4]. These biological properties likely contribute to the symptomatic improvements observed in the present cohort.

A key objective of this study was to clarify whether chronological age influences treatment response after SVF injection. Our results demonstrated no significant association between age and either baseline or final VAS scores, and age was not correlated with other baseline characteristics. This suggests that older patients can achieve pain relief comparable to that of younger patients following SVF therapy. Historically, age has been considered a potential negative prognostic factor in regenerative medicine because aging is associated with reduced stem cell proliferative capacity and impaired tissue healing [15,16,25,26]. However, the clinical relevance of chronological age in SVF therapy has remained unclear. Our findings align with emerging clinical evidence suggesting that patient age alone may not be a reliable predictor of outcomes in biologic treatments for knee OA. One possible explanation is that SVF represents a heterogeneous cell population with strong paracrine and immunomodulatory activity, which may mitigate age-related declines in individual stem cell function. In addition, the autologous and minimally manipulated nature of SVF may preserve sufficient regenerative capacity even in older individuals. Collectively, these mechanisms may explain why chronological age did not significantly influence clinical improvement in the present study. Clinically, this finding is important because knee OA predominantly affects older patients, and unnecessary age-based exclusion could limit access to potentially beneficial regenerative therapies.

In contrast to age, BMI demonstrated a significant association with final pain outcomes in this cohort. Patients with higher BMI exhibited worse residual pain at final follow-up, indicating that obesity may negatively influence the therapeutic response to SVF. This observation is consistent with previous literature. Zhang et al. [27] reported that BMI was an independent predictor of prognosis after SVF treatment, with higher BMI associated with poorer clinical outcomes. The adverse effect of obesity on knee OA is likely multifactorial. First, increased mechanical loading across the knee joint in overweight patients may accelerate cartilage degeneration and limit symptomatic improvement [28,29]. Second, obesity is associated with a chronic low-grade inflammatory state characterized by elevated adipokines and pro-inflammatory cytokines, which may counteract the anti-inflammatory effects of SVF [30,31]. Third, metabolic dysregulation in obese individuals may impair the joint microenvironment and reduce regenerative responsiveness [32,33]. Taken together, these mechanisms may explain why BMI emerged as a more important clinical determinant than chronological age in the present study.

Radiographic severity based on K–L grade was also significantly associated with pain outcomes. Patients with more advanced OA (grade IV) showed worse baseline and follow-up VAS scores compared with those with less severe disease. This finding is biologically plausible and consistent with prior studies indicating that structural joint damage influences the magnitude of response to regenerative therapies. Advanced cartilage loss, subchondral bone changes, and joint deformity may limit the capacity for biological treatments to fully restore joint homeostasis. Therefore, although SVF therapy can provide symptomatic benefit across disease stages, earlier intervention before severe structural deterioration may yield more favorable outcomes.

Another important finding of this study was the significant association between the number of injected SVF cells and final pain scores. Patients receiving higher cell counts demonstrated better clinical outcomes, suggesting a potential dose–response relationship. This observation is consistent with prior work demonstrating that cellular factors can influence the efficacy of biologic therapies for knee OA. Given that SVF exerts its effects largely through paracrine signaling and immunomodulation, a greater number of viable cells may enhance the therapeutic milieu within the joint [4,7,9]. From a clinical perspective, these findings support the importance of optimizing cell yield and standardizing SVF preparation protocols to maximize treatment efficacy.

The present study provides several clinically relevant insights. First, chronological age alone should not be considered a contraindication for SVF therapy. Second, BMI and structural disease severity appear to be more important determinants of outcome. Third, adequate SVF cell dose may enhance clinical response. These findings may help refine patient selection and support a more personalized approach to regenerative treatment in knee OA.

Interestingly, although advancing age has traditionally been viewed as a potential disadvantage in regenerative therapies, older patients may possess certain practical advantages in the context of SVF harvesting. Aging is commonly associated with an increase in total body fat mass and adipose tissue distribution [34,35]. This may allow the procurement of a larger volume of lipoaspirate in elderly individuals compared with younger patients. Because the therapeutic efficacy of SVF is partly dependent on the number of viable cells delivered, a greater harvestable adipose volume may translate into a higher SVF cell yield. In the present study, the number of injected SVF cells was significantly associated with improved clinical outcomes, suggesting a dose-dependent therapeutic effect. From this perspective, the ability to obtain a larger adipose volume in older patients could represent a potential advantage rather than a limitation. Therefore, chronological age alone should not be regarded as a negative selection factor for SVF therapy and, in certain cases, older patients may achieve favorable outcomes when adequate cell numbers are delivered. Nevertheless, the relationship between age, adipose tissue characteristics, and functional SVF cell potency remains complex. While increased adiposity may facilitate higher cell yield, age-related cellular senescence and variability in SVF composition may still influence biological activity. Accordingly, further studies evaluating both quantitative and qualitative aspects of SVF in different age groups are warranted.

This study has several limitations. First, the retrospective design may have introduced selection bias and limits the ability to establish causality. Second, the follow-up period was limited to 12 months; therefore, the long-term durability of the clinical response, including outcomes beyond 2 years, remains uncertain. Third, this study did not include a placebo, sham, or active control group, and therefore the observed improvement cannot be attributed exclusively to SVF treatment. A prospective randomized controlled trial comparing SVF with placebo or standard nonoperative treatment would provide stronger evidence. Fourth, although age, BMI, radiographic severity, and injected SVF cell number were evaluated, other potential confounders such as physical activity level, alignment, muscle strength, metabolic status, concomitant medications, and patient expectations were not fully assessed. In addition, multivariable regression analysis was not performed, and therefore the independent effects of BMI, structural disease severity, and SVF cell dose on treatment response could not be fully determined. Fifth, clinical outcome assessment was mainly based on VAS pain scores, and validated functional outcome measures such as Western Ontario and McMaster Universities Arthritis Index or Knee injury and Osteoarthritis Outcome Score were not included. Finally, because some patients contributed both knees to the analysis, statistical independence may have been partially violated. Future prospective studies with longer follow-up, appropriate control groups, and multivariable or mixed-effects statistical models are required to confirm the independent predictors of response to SVF therapy.

## 5. Conclusions

In this retrospective cohort, intra-articular autologous adipose-derived SVF injection was associated with significant 12-month pain reduction in patients with knee OA. Chronological age was not significantly associated with clinical outcome, whereas BMI, radiographic OA severity, and injected SVF cell number were more closely related to final pain status. Because of the retrospective design, lack of a control group, and limited follow-up duration, these findings should be interpreted as hypothesis-generating and require confirmation in prospective controlled studies.

## Figures and Tables

**Table 1:** Baseline characteristics.

Age, y	63.6 ± 7.5 (43–86)
Sex, male/female, *n*	111/246
Side of involvement, right/left, *n*	172/185
Body mass index, kg/m^2^	25.7 ± 3.2 (18.9–32.1)
Kellgren-Lawrence grade, *n* (%)	
II	143 (40.1)
III	149 (41.7)
IV	65 (18.2)

Data are presented as means ± standard deviation (range) unless otherwise indicated. Distributions of sex, side of involvement and Kellgren-Lawrence grade were calculated per knee.

**Table 2:** Pain scores stratified by characteristics.

	VAS			
	Initial	*p* Value	Final Follow-Up	*p* Value
Age, y		0.128		0.088
<50 (*n* = 11)	7.2 ± 0.9		2.7 ± 0.6	
50–60 (*n* = 118)	6.5 ± 1.3		3.0 ± 1.6	
60–70 (*n* = 152)	6.6 ± 1.2		3.1 ± 1.5	
70–80 (*n* = 67)	6.7 ± 1.1		3.7 ± 1.7	
≥80 (*n* = 9)	6.4 ± 1.5		3.8 ± 2.0	
Sex		0.218		0.075
Male (*n* = 111)	6.4 ± 1.3		2.9 ± 1.5	
Female (*n* = 246)	6.6 ± 1.3		3.3 ± 1.6	
Side of involvement		0.138		0.477
Right (*n* = 172)	6.5 ± 1.2		3.2 ± 1.6	
Left (*n* = 185)	6.7 ± 1.3		3.2 ± 1.6	
Body mass index, kg/m^2^		0.247		<0.001
<20.0 (*n* = 14)	6.6 ± 1.2		3.4 ± 1.8	
20.0–24.9 (*n* = 135)	6.6 ± 1.3		2.8 ± 1.3	
25.0–29.9 (*n* = 152)	6.5 ± 1.2		3.1 ± 1.6	
≥30.0 (*n* = 56)	6.9 ± 1.2		4.2 ± 1.9	
Kellgren–Lawrence grade		0.003		0.001
II (*n* = 143)	6.3 ± 1.2		2.9 ± 1.5	
III (*n* = 149)	6.6 ± 1.2		3.2 ± 1.6	
IV (*n* = 65)	6.9 ± 1.3		3.8 ± 1.7	
Number of SVF, ×10^7^		0.716		0.004
<7.0 (*n* = 94)	6.6 ± 1.2		3.7 ± 1.7	
7.0–8.0 (*n* = 236)	6.6 ± 1.3		3.0 ± 1.5	
≥8.0 (*n* = 27)	6.3 ± 1.1		2.9 ± 1.8	

VAS, visual analog scale; K–L, Kellgren–Lawrence; SVF, stromal vascular fraction.

**Table 3:** Correlations between patient age and baseline characteristics.

	Age	
	S rho	*p *Value
Sex	0.097	0.066
Side of involvement	0.046	0.384
Body mass index	0.090	0.089
Kellgren–Lawrence grade	−0.016	0.770
Number of SVF	−0.064	0.225

Calculated using the Spearman rank-order test. S, Spearman; SVF, Stromal vascular fraction.

## Data Availability

The data presented in this study are available on request from the corresponding author.
